# A constructivist-based proposal for bioinformatics teaching practices during lockdown

**DOI:** 10.1371/journal.pcbi.1008922

**Published:** 2021-05-13

**Authors:** Cristóbal Gallardo-Alba, Björn Grüning, Beatriz Serrano-Solano

**Affiliations:** Bioinformatics Group, Department of Computer Science, Albert-Ludwigs-University Freiburg, Freiburg, Germany; University of Toronto, CANADA

## Abstract

The Coronavirus Disease 2019 (COVID-19) outbreaks have caused universities all across the globe to close their campuses and forced them to initiate online teaching. This article reviews the pedagogical foundations for developing effective distance education practices, starting from the assumption that promoting autonomous thinking is an essential element to guarantee full citizenship in a democracy and for moral decision-making in situations of rapid change, which has become a pressing need in the context of a pandemic. In addition, the main obstacles related to this new context are identified, and solutions are proposed according to the existing bibliography in learning sciences.

## Introduction

The Coronavirus Disease 2019 (COVID-19) pandemic has caused serious alterations in the world’s education system, and due to the viral characteristics, it is likely that this situation will repeat in the future. This reality has forced a crash course for online learning plans and technology for students and faculty members.

Universities across the globe have been compelled to alter their functioning in order to adapt teaching and research activities to the new context. In the words of Carol McQuiggan, former Director of the Faculty Center for Teaching and Instructional Technology at Penn State Harrisburg, “what worked for them in the past in their traditional classroom may no longer be helpful or reliable in the distance education context” [1].

Qualitative research into online learning suggests that students experience greater dissatisfaction, interpersonal isolation, feelings of unclear direction and uncertainty, and a lack of engagement in this environment [2–5]. Despite not being associated with a single cause, research indicates that a crucial mistake is to perceive technology only as a channel for transferring content, used as a substitute for other tools, ignoring the growing knowledge about pedagogical practices in online education [6,7].

The results of several studies indicate that the constructivist learning theory is the most appropriate to exploit the potential of technology-mediated educational practice, an intrinsic feature of distance education [8]. Constructivism considers that the best way to acquire knowledge is through active experimentation in real contexts which encourage the selection, organization, and integration of their experiences with their previous knowledge in a social context [9,10]. Due to its social nature, the role of technology in constructivist online teaching practices should be the creation of learning environments focused on the collective construction of knowledge rather than simply a vehicle for the delivery of content [11].

This paper summarizes the pedagogical foundations necessary to develop quality distance bioinformatics education practices, according to the existing bibliography. We propose different tools, such as GitHub and the Galaxy platform for bioinformatics data analysis, as example frameworks to make those recommendations more illustrative (see Box 1 for practical tips). It will also set the ground for trainers to organize their bioinformatics lessons by establishing the theoretical basis for our recommendations on online teaching described in Serrano-Solano and colleagues [11].

**Box 1. Summary of practical tips for instructors of bioinformatics**

- Make the learning process active and dynamic: Let students experience rather than be passive learners by boosting discussion and reflection.
- Keep the motivation up: Use real-life problems, and understand the student’s short- and long-term personal learning goals.
- Be clear: Give direct instructions in a precise and transparent way.
- Be flexible: Let students control the pace of learning starting from certain guidelines, allow them to self-organize the learning process.
- Avoid hierarchical media: Bidirectional communication promotes critical thinking.
- Use synchronous channels to foster social presence.
- Use asynchronous channels for higher levels of thinking.
- Build a community: Be close, create a cooperative environment that encourages constructive discussion, and share and challenge ideas to promote social interaction.

## A constructivist approach to bioinformatics education

Constructivism roots have their origin in the work of the Swiss psychologist Jean Piaget. Its core idea is that knowledge acquisition is a dynamic process which must be led by the learner through experience, discussion, and reflection [12,13]. It assumes that students learn better when they control the pace of learning, giving a special value to learning experiences which involve real-life problems [14–16]. According to this paradigm, students are not passive recipients of information, but they must act as the active protagonist of the learning process [17].

Social constructivism emphasizes that the knowledge construction process takes place more efficiently in a social context, where ideas can be shared and challenged [18,19]. This is in concordance with research by Palloff and Pratt, which points out that establishing a learning community is essential for implementing online learning practices successfully [20].

A concept closely associated with social constructivism is social presence. It is defined as the competence to transmit the feeling of closer social contacts in a certain communicative context and considered an essential factor for establishing functional learning communities [21,22]. According to this construct, the potential of certain communication technology to transmit social presence is directly proportional to its ability to transmit nonverbal information [23].

Although the constructivist theory has profoundly influenced the teaching of science and mathematics, its applicability to computer science–related fields has received much less attention. A few recommended examples of publications that have analyzed computer sciences educational practices from a constructivist point of view include Ben-Ari (2001), Chesnevar and colleagues (2004), Connolly (2006), and Feliciano (2015).

Bioinformatics education can be defined as the development and application of computational methods to collect, store, interpret, and integrate data in order to solve biological problems [24,25]. According to Magana and colleagues (2014), it includes 4 main dimensions: conceptual, methodological, computational tool management, and information resources [26]. For the purpose of this article, the last 2 categories have been grouped, since we consider that through the knowledge of computational tools it is possible to acquire the necessary skills to manage existing databases.

### Conceptual dimension

The conceptual dimension refers to how to teach the abstract ideas or general notions that any student must know and understand in order to be able to solve biological problems.

According to the constructivist paradigm, the acquisition of new knowledge is a recursive process in which new concepts are built on previous ones. It also states that the learning process of highly abstract concepts is not possible if there is no previous conceptual foundation [9,27]. It explains that one of the first challenges facing bioinformatics students is the lack of viable mental models of basic elementary computer science or biological concepts, which usually leads to frustration. It is the responsibility of the teacher to identify common misconceptions, and from that, to guide the students in the construction of the conceptual foundation which capacitate them to develop their learning process autonomously [28,29]. The use of asynchronous communication tools in which students can discuss, such as forums, has proven to be one of the most useful methods for improving conceptual understanding, allowing collective conceptual construction [30,31].

On the other hand, diverse authors agree that to implement adequate online teaching practices, it is necessary to take into account the characteristics of student’s learning styles, as it allows instructors to infer which formats are most suitable to facilitate the acquisition of new mental models [29,31–33]. Thus, students with an abstract learning model tend to identify more easily the patterns inherent in an abstract model, whereas concrete learners prefer metaphors or analogies to facilitate learning new conceptual structures [29,34]. For this reason, to ensure that education is student centered, it is essential that educational resources include diverse formats to address differences in learning styles and learning paces [35,36].

Learning Management Systems (LMS) have become one of the essential tools in distance education practices by providing a supportive learning environment, facilitating the creation and distribution of educational content and communication tools [37]. According to an analysis conducted by EMBL-EBI, Drupal, Moodle, and ATutor are the most suitable platforms for the implementation of bioinformatics training programs [38]. However, despite being useful tools for conceptual teaching, LMS are often oriented toward administrative purposes and have a limited potential for implementing cooperative learning based on real-life challenge-based problems [39–41].

### Methodological dimension

The methodological dimensions, usually referred to as computational biology, includes the analysis, design, implementation, and evaluation of computational processes for modeling biological phenomena. Due to its strong logical and algorithmic structure, it is useless to base its teaching simply on memorization [42]. The set of skills needed in this area, such as the capacity for multilevel abstraction or reduction and decomposition in solving complex problems, requires that students adopt a metacognitive framework known as computational thinking [43,44]. It can be defined as the ability to recognize computational aspects in natural processes which configure our reality and apply the tools and techniques of computing to understand and model them [44].

Teaching creative programming, i.e., to use programming for solving ill-defined problems, is considered a suitable approach to encourage computational thinking, allowing to develop algorithmic thinking, problem-solving, logic, and debugging skills [45,46]. However, the constructivist theory suggests that before proceeding to teach programming, it is necessary for students to possess adequate computer sciences conceptual models [28].

From a constructivist point of view, collaborative problem-based learning is considered the most adequate pedagogic strategy for teaching experimental, statistical, and computational processes, since it allows students to actively participate in the construction of their knowledge through social interaction by resolving real problems [47–50]. In addition, collaborative learning methods have proven to be very useful in encouraging critical thinking, ensuring a higher level of learning [50,51].

Several studies have shown the usefulness of Git combined with the hosting service GitHub as an educational tool in the field of computer science for the development of methodological skills [52–54]. Among the reasons that have determined its success as a collaborative learning tool is the fact that it is the de facto platform for open-source projects, allowing the students to collaborate in real-life scenarios [39,52]. One of the most interesting features to facilitate user collaboration is the Pull Request mechanism, which allows to request, review, and discuss changes to be made in the content of a project or even in the course content, enabling a participatory culture [39,55,56]. Although GitHub was not designed as an LMS, in recent years, multiple tools have been developed to complement its functionality. One such tool is GitHub classroom, which facilitates the distribution of repositories and the organization of working groups through the use of a web interface [52,57].

Rosalind (http://rosalind.info/problems/locations/) is another resource that has proven very useful for teaching methodological skills through a problem-based approach [58]. One of the most interesting aspects of this platform is that it allows problems to be automatically corrected, a feature that favors autonomous learning.

### Computational tools management and information resources dimension

This category includes the knowledge of preexisting instruments and applications and the ability to use them to solve biological problems. One of the difficulties associated with this dimension of learning is that setting up a suitable environment for effective bioinformatics analysis can be challenging for users without the technical knowledge to manage a compute infrastructure [59].

The cognitive load theory describes learning as the result of sequential processing of information, which involves 3 types of memory: immediate memory, working memory, and long-term memory [60]. According to this theory, since the working memory has a limited capacity, adequate learning resources are required to avoid overloading it with activities that don’t contribute to learning, due to which it is necessary that the computational tools can be easily used, linked to each other, and maintained [61].

The Galaxy project (https://galaxyproject.org) provides a framework which enables students without programming and system administration competences to perform computational analysis through the web browser [62]. It includes thousands of bioinformatic tools, integrated into an interface that allows connecting tools, setting tool parameters, and sharing datasets, histories, workflows, and visualizations [62]. From a constructivist perspective, in order to take advantage of the educational potential that Galaxy offers, it is necessary to encourage students to share the analysis results, as well as to stimulate discussion and reflection on those results, promoting the acquisition of new knowledge as a result of the interaction within the learning community.

One of the features that make Galaxy a suitable tool for distance education is that it provides container-based frameworks for creating portable Galaxy instances, allowing to carry out analysis in situations where no internet connection is available [63]. This platform, originally conceived as a research tool, integrates a community-driven teaching framework with a wide collection of training materials covering diverse bioinformatics domains [63,64]. Some practical recommendations for using Galaxy as an e-learning platform have been compiled in Serrano-Solano and colleagues [11].

ORCA and BioLinux are other tools developed in recent years that have proven useful in the field of bioinformatics education. ORCA provides hundreds of popular bioinformatics tools and their dependencies in a containerized environment [59]. BioLinux is an Ubuntu-based distribution which includes more than 250 preinstalled software packages, providing a portable and integrated environment for bioinformatics analysis [65]. However, unlike Galaxy, both require basic command line knowledge.

## Major online learning challenges

As claimed by Goolam Mohamedbhai, member of the governing council of the United Nations University, “it is a fallacy to believe that online learning can be effective by merely posting a lecturer’s notes online or having a video recording of the lecture” [66]. In addition, the performance of the learning community can be affected if the majority of the members do not manage to adapt to the online context [67]. Therefore, it is important to carefully analyze the e-learning’s problematic dimensions.

### Teaching effectiveness in technology-mediated learning

Technological tools, due to their design, do not act just as neutral means for transmitting information, but they also transmit values and habits of thought [68,69]. Thus, for example, when teaching is dominated by 1-way media, hierarchical relationships are promoted, which entails an attack on critical thinking [70].

When considering video recordings as teaching tools, it is important to incorporate those auxiliary technologies which could increase their pedagogical potential, such as including short quizzes [71]. Those strategies can result in improved teaching and social presence, which, in turn, are linked to an increase in the students’ engagement.

Communication is crucial for assessment but a critical point to take into account when selecting the teaching tools is that synchronous and asynchronous ones should be used for different educational purposes in online courses. Research indicates that synchronous discussions are more useful for fostering social presence, while asynchronous communication for developing higher levels of thinking [72,73].

### Teacher’s role in online teaching

Web-based environments require deep cultural shifts, such as sharing control of the learning process, which can result in a loss of teacher’s professional identity, usually linked to higher level of stress [74–77].

Results indicate that, in a video-conferencing environment, an instructor’s positive attitude toward technology and interactive teaching style are related to perceived learning effectiveness, with teaching style showing the most important influence on student involvement and participation [78]. Teachers who are skilled in community building are considered particularly valuable [79]. Qualitative data suggest that students place a high priority on the instructor’s ability to establish and maintain an engaging and constructive discussion environment, with interactive activities playing a major role in enhancing learning and motivation [80–82].

### Student engagement in online environments

Online learning puts special demands on students to stay motivated and focused [83,84]. For this reason, instructors must consciously supply this need with a combination of motivational techniques.

Firstly, as in classroom teaching, intrinsic motivation is key. To boost it, instructors ideally need to understand each student’s short and long-term personal learning goals and then design activities that resonate with them, if possible by using real-life problems [83,85].

Another motivational technique which has been suggested as extremely useful consists of fostering learner’ self-directed learning pace [85]. To this end, it is essential to establish a cooperative environment in which students were able to self-organize the learning process, starting from certain guidelines provided by the instructor, who should act as a facilitator rather than as an authority on the subject [41,86–88].

Finally, a third motivational technique considered useful for improving teaching effectiveness and student engagement is the design of learner-centered syllabus, characterized by shared decision-making and structured and clear objectives [89,90].

### Creating an effective learning community

Establishing a functional learning community has been proved to be a key factor for overcoming some of the major drawbacks linked to distance education, such as the student’s feeling of isolation and their lack of motivation and interaction [91–93].

It is the instructor’s responsibility to stimulate the creation and maintenance of a structured network of reciprocal interactions between students that enables the collective construction of knowledge efficiently [94]. Research results indicate that students who are appropriately integrated into learning communities obtain higher grades [95]. Quantitative results suggest a relationship between interaction levels and group size and that group size is a critical factor to the effectiveness of learning [96,97]. This is especially important in the context of collaborative learning platforms, as small groups allow students to feel that their contributions are more important to the success of the project [98]. One of the strategies that can facilitate work in small groups is peer learning, through which students learn by teaching their peers [99].

### Adapting teaching models to students requirements

One of the aspects to consider at the moment of implementing practices of distance education is how the chosen methods affect the digital inequality. Recent studies indicate that low-income students experienced substantially greater reductions in learning performance relative to high-income students [100]. One of the main concerns identified is the instability of the network, which makes it difficult to establish synchronous communication channels [101]. In order to alleviate these difficulties, it is essential that educational materials are available to students in an asynchronous way, such as video or audio recordings.

The neurodiversity and cultural differences of the students are additional aspects to take into account at the time of designing a course, given that many of the difficulties they experience in classroom teaching can be increased in the case of distance education. Thus, for example, in the case of the presence of students with Asperger’s syndrome, it is recommended that educational materials allow for the adaptation of font size, as well as the use of colors [102].

## Conclusions

The COVID-19 pandemic is probably going to entail a turning point for the global educational system. The profound cultural changes imposed by the general situation of uncertainty are going to force the previous teaching practices to adapt to this new context.

We consider that, in order to collectively face the challenges associated with the current situation, it is necessary to establish a conceptual framework shared by the whole educational system and capable of offering the necessary tools to uphold the quality of the educational practices.

Our proposal relies on a fundamental pillar: the constructivist paradigm as the philosophy of learning, that is, to consider the promotion of autonomous thinking as an essential element to guarantee full citizenship in a democracy and for moral decision-making in situations of rapid change. Assuming this theoretical foundation, it is possible to overcome the main caveats of distance education—such as the student’s feeling of isolation or the teacher’s ability to maintain their engagement in online environments—by paying special attention to the roles of technology, teaching practices, learning communities, motivation, and the way these elements interact with each other.
